# 16S gut community of the Cameron County Hispanic Cohort

**DOI:** 10.1186/s40168-015-0072-y

**Published:** 2015-03-06

**Authors:** Matthew C Ross, Donna M Muzny, Joseph B McCormick, Richard A Gibbs, Susan P Fisher-Hoch, Joseph F Petrosino

**Affiliations:** Alkek Center for Metagenomics and Microbiome Research, Baylor College of Medicine, Houston, TX USA; Department of Molecular Virology and Microbiology, Baylor College of Medicine, Houston, TX USA; Human Genome Sequencing Center, Baylor College of Medicine, Houston, TX USA; University of Texas School of Public Health, Brownsville, TX USA

## Abstract

**Background:**

Obesity and type 2 diabetes (T2D) are major public health concerns worldwide, and their prevalence has only increased in recent years. Mexican Americans are disproportionately afflicted by obesity and T2D, and rates are even higher in the United States-Mexico border region. To determine the factors associated with the increased risk of T2D, obesity, and other diseases in this population, the Cameron County Hispanic Cohort was established in 2004.

**Results:**

In this study, we characterized the 16S gut community of a subset of 63 subjects from this unique cohort. We found that these communities, when compared to Human Microbiome Project subjects, exhibit community shifts often observed in obese and T2D individuals in published studies. We also examined microbial network relationships between operational taxonomic units (OTUs) in the Cameron County Hispanic Cohort (CCHC) and three additional datasets. We identified a group of seven genera that form a tightly interconnected network present in all four tested datasets, dominated by butyrate producers, which are often increased in obese individuals while being depleted in T2D patients.

**Conclusions:**

Through a combination of increased disease prevalence and relatively high gut microbial homogeneity in the subset of CCHC members we examined, we believe that the CCHC may represent an ideal community to dissect mechanisms underlying the role of the gut microbiome in human health and disease. The lack of CCHC subject gut community segregation based on all tested metadata suggests that the community structure we observe in the CCHC likely occurs early in life, and endures. This persistent ‘disease’-related gut microbial community in CCHC subjects may enhance existing genetic or lifestyle predispositions to the prevalent diseases of the CCHC, leading to increased attack rates of obesity, T2D, non-alcoholic fatty liver disease, and others.

**Electronic supplementary material:**

The online version of this article (doi:10.1186/s40168-015-0072-y) contains supplementary material, which is available to authorized users.

## Background

Obesity is a major public health problem worldwide and the number one risk factor associated with multiple diseases, including type 2 diabetes (T2D). T2D is currently the most prevalent endocrine disease in the world and is estimated to afflict 430+ million people by 2030 [1]. T2D is a multifactorial disorder, with pathogenic contributions from genetics, the environment, and lifestyle [2,3]. Accumulating evidence shows metabolic diseases like T2D develop because of chronic, low grade, systemic inflammation that leads to disruption of the normal gut microbiota [4]. Recent studies have revealed gut microbiome signatures associated with obesity and T2D patients [5,6]. Two independent studies in European and Chinese populations revealed increased abundances of opportunistically pathogenic *Clostridium* species and decreased abundances of butyrate-producing *Roseburia*, *Faecalibacterium*, and *Eubacterium* species associated with T2D patients [7,8]. Karlsson *et al*. [7] also found increased abundances of *Lactobacillus gasseri* and *Streptococcus mutans* predictive of insulin resistance while Qin *et al*. [8] found enrichment in *Escherichia coli* associated with current T2D patients. There is mounting evidence that the structure of the gut microbial community has significant implications for health and disease, and therapeutic manipulations of these communities can have immediate effects [9-14]. For example, in mice, deficiency of Toll-like receptor 5 (TLR-5), an innate immune sensor of flagellin, results in mice that mimic the symptoms of metabolic syndrome including hyperlipidemia, hypertension, and insulin resistance [15]. These symptoms were induced in wild-type germ-free mice upon fecal transfer from the TLR-5 deficient donors. Likewise, there is evidence that direct alteration of the gut microbiome through fecal transplantation can temporarily reverse many of the symptoms associated with metabolic syndrome and other diseases including ulcerative colitis, irritable bowel syndrome, and chronic fatigue syndrome [12,14,16]. Diet can have a major impact on the composition of the gut microbial community and has been implicated in the establishment of gut enterotypes, which are distinct microbial community signatures driven by the differential abundance of certain key taxa [17]. However, once established, enterotype-defining taxa appear to be resistant to modification through dietary intervention [18], suggesting that events early in life establish enduring signatures in the gut microbial composition [19]. However, studies in animals and humans implementing dietary interventions aimed at improving metabolic markers have noted non-enterotype-defining shifts in the gut microbiome associated with improved health [13,20-23]. For example, *Akkermansia muciniphila*, a closely adherent mucin-degrading bacterium, is depleted after administration of a high-fat diet and is strongly negatively associated with obesity and T2D [24]. Dietary supplementation of mice with *A. muciniphila* reversed the high-fat-diet effects, such as inflammation and insulin resistance, presumably through improved gut barrier function. As a result, there is mounting enthusiasm for altering the microbiome through changes in lifestyle, diet and/or probiotics to help prevent and alleviate many of the disease risks associated with obesity and T2D.

Americans of Mexican descent (MAm) are at an increased risk of obesity and T2D compared to all Americans (Am) nationally (MAm 39.1% BMI ≥30, T2D 12.8%) (Am 35.7% BMI ≥30, T2D 8.3%) [25-27]. Diabetes in Hispanics occurs earlier (mean age of diagnosis 49.4 years for Hispanics, 53.8 years for all Americans), manifests with higher complication rates, and attacks at nearly twice the rate of non-Hispanic whites (WAm) (T2D 12.8% MAm, T2D 7.6% WAm [28]). Mexican Americans living along the United States (US)-Mexico border are at even greater risk of developing T2D compared to Mexican Americans nationally (15.7% along the border vs. 12.8% nationally) [29-33]. This likely has roots in the genetic makeup of this population, lifestyle, diet, and socioeconomic status among other factors.

**Figure 1:**
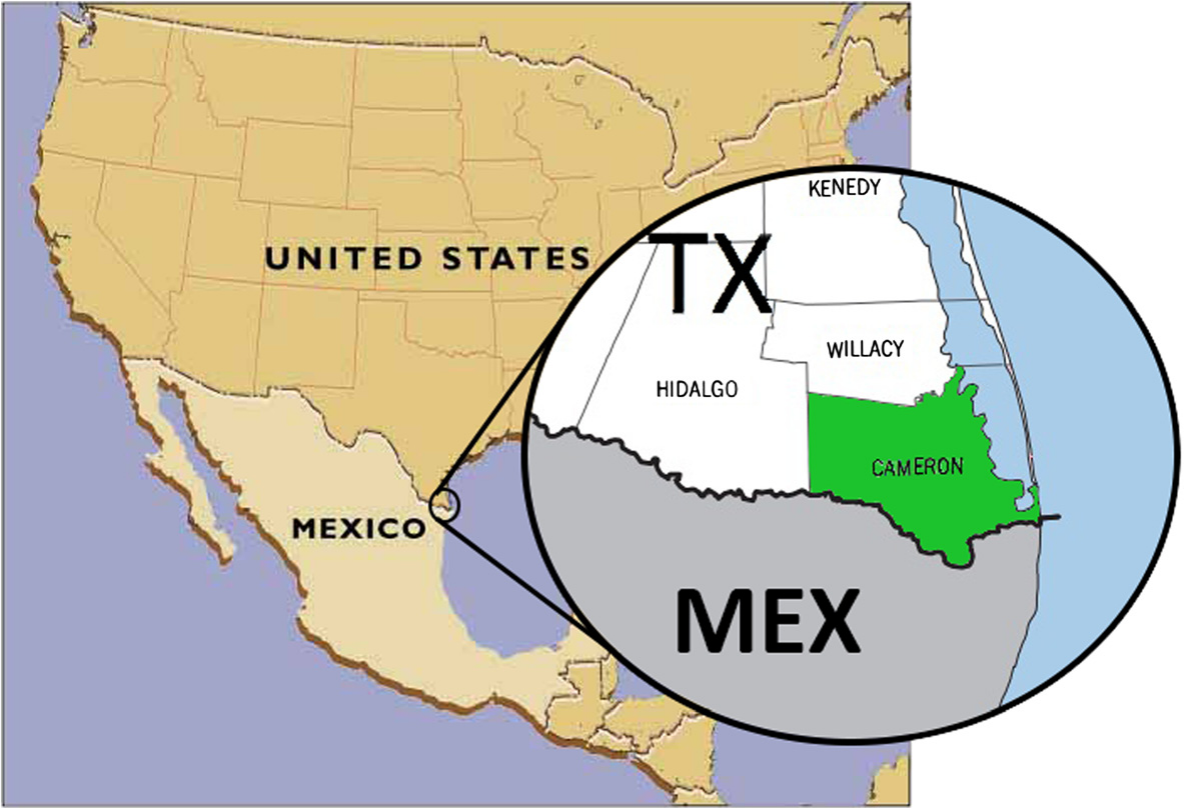
**Location of Cameron County, Texas.** MEX, Mexico; TX, Texas.

**Figure 2:**
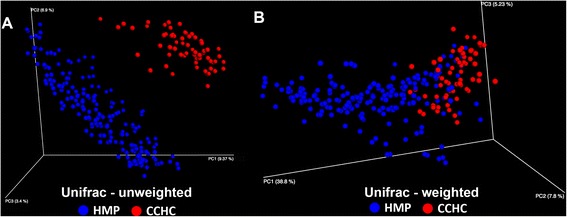
**16S rRNA sequence principle coordinates analysis (PCoA) plots of CCHC and HMP stool samples. (A)** Comparison of stool samples from the CCHC and HMP analyzed using the unweighted UniFrac metric. **(B)** Same stool samples as in 1A analyzed using the weighted UniFrac metric; HMP *n* = 213, CCHC *n* = 63. CCHC, Cameron County Hispanic Cohort; HMP, Human Microbiome Project; PC, principal coordinate.

**Figure 3:**
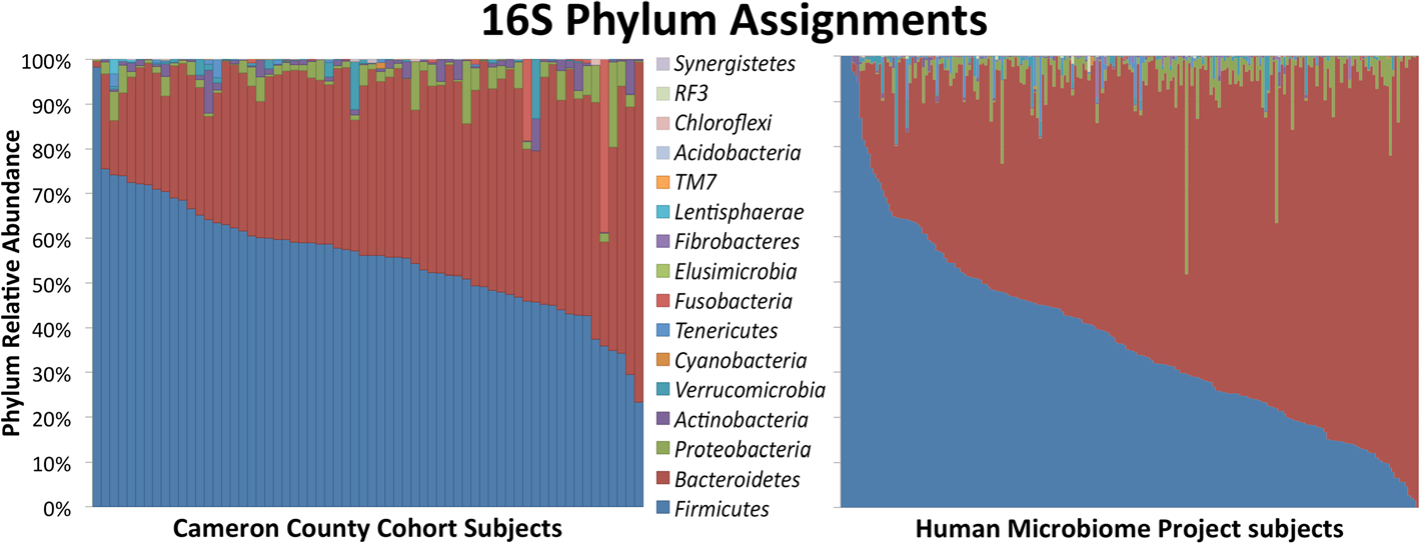
**Phylum level analysis of CCHC and HMP subject stool samples.** HMP *n* = 213, CCHC *n* = 63.

The US-Mexico border region comprises a diverse mixture of economies and disease burdens owing to the very unique countries that lie on either side. Driving this dichotomy is the greater than fivefold disparity between the GDP-per-capita of these two countries [34]. To identify the important risk factors for obesity and T2D of Mexican Americans living in the lower Rio Grande Valley, the Cameron County Hispanic Cohort (CCHC) was established in 2004 [35] (Figure 1). Overall, members of this community have much higher obesity (50.9% BMI ≥30, 9.0% BMI ≥40) and T2D rates (28.0%) versus the average American population (35.7% BMI ≥30, 6.3% BMI ≥40, T2D 8.3%) [36-38]. These statistics reflect the general trend of Mexican Americans living along the entire US-Mexico border [27]. Participants in this study were slightly heavier but had lower T2D rates than the CCHC as a whole (60% BMI ≥30, 12.9% T2D). The CCHC represents the first exclusively Mexican American group from a border city with poor overall health. In light of the previously described recent studies, and the disproportionately increased prevalence of T2D and obesity in this population, we sought to characterize the gut microbiome in 63 subjects belonging to the CCHC. For this study, we chose to utilize Human Microbiome Project (HMP) stool data for comparative analysis [39-41] (Figure 2) (Figure 3). The 300 participants of the HMP were subjected to a lengthy list of exclusion criteria and here represent a healthy Western microbiome [42]. Using the HMP stool data as a reference will help determine associations with the gut microbial structure and increase our understanding about the observed predisposition to obesity and T2D in the CCHC.

## Results and discussion

### 16S rRNA gene sequencing

16S rRNA gene profiling of 63 CCHC subjects revealed that no taxa is significantly associated with any clinical measure evaluated in this study (after correcting for multiple comparisons). These included BMI, age, cholesterol, waist-to-hip ratio, diabetes status, triglycerides, sex, fasting glucose, and others (complete metadata included in Additional file 1). We found this apparent high level of homogeneity across subjects surprising, as the variance between subjects of the CCHC was about one third of that observed between HMP subjects.

**Figure 4:**
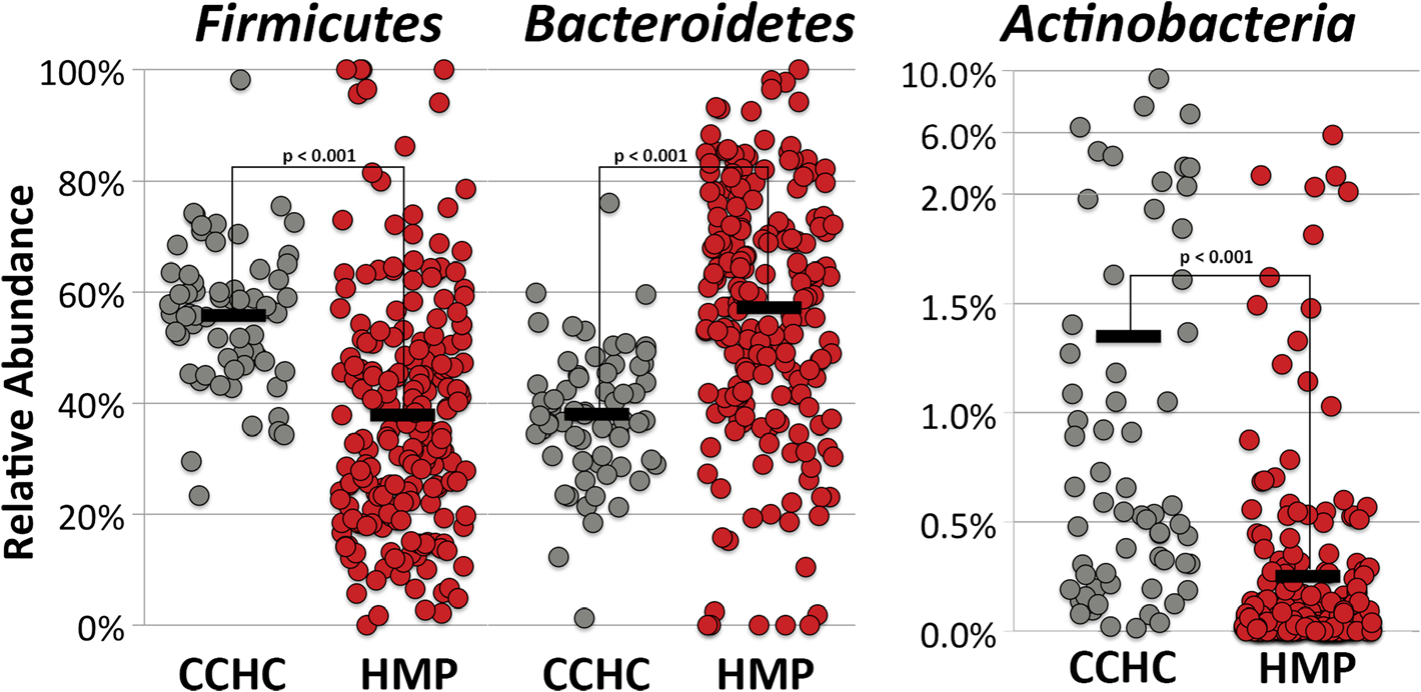
**Breakdown of phylum level analysis showing the three most abundant phyla.** Scatter plots representing 16S rRNA relative abundance of the three most prevalent phyla: *Firmicutes*, *Bacteroidetes*, and *Actinobacteria*. Black bars represent mean relative abundance. All *P* values were calculated by Mann-Whitney *U* test. HMP *n* = 213, CCHC *n* = 63. CCHC, Cameron County Hispanic Cohort; HMP, Human Microbiome Project.

In the CCHC subjects, we found significantly elevated levels of organisms belonging to the *Firmicutes* (*P* < 0.001, FDR <2%) and *Actinobacteria* (*P* < 0.001, FDR <2%) phyla, while there were significantly fewer *Bacteroidetes* (*P* < 0.001, FDR <2%) compared to subjects in the HMP (Table 1) (Figure 4). At the family level, the CCHC showed significantly increased *Lachnospiraceae* (*P* < 0.001, FDR <1%), *Veillonellaceae* (*P* < 0.001, FDR <1%), *Coriobacteriaceae* (*P* < 0.001, FDR <1%), *Ruminococcaceae* (*P* < 0.002, FDR <1%), and *Prevotellaceae* (*P* < 0.005, FDR <2%) compared to HMP subjects. Significantly decreased families include *Bacteroidaceae* (*P* < 0.001, FDR <1%) and *Rikenellaceae* (*P* < 0.001, FDR <1%). For those operational taxonomic units (OTUs) assignable to a genus, many of the significant differences occur in genera previously reported as associated with obesity and/or T2D. The groups of significantly increased genera in the CCHC include *Prevotella* (*P* < 0.003, FDR <4%), *Collinsella* (*P* < 0.001, FDR <2%), *Roseburia* (*P* < 0.001, FDR <2%), *Streptococcus* (*P* < 0.001, FDR <2%), and *Dialister* (*P* < 0.003, FDR <4%). Those significantly decreased in the CCHC include *Bacteroides* (*P* < 0.001, FDR <2%), *Alistipes* (*P* < 0.001, FDR <2%), and *Parabacteroides* (*P* < 0.001, FDR <2%). Many differences between the CCHC gut microbial community and HMP subjects noted above tend to mirror shifts often observed in studies comparing subjects who are obese or have T2D versus healthy controls. However, while T2D and obesity are pervasive in the CCHC, many members who were examined in this study are not obese or diabetic (40% BMI <30, 87.3% HbA1c <6). This suggests that the gut microbiome may be a contributing factor to the development of metabolic disease or that the microbial community structure may serve as a predictive biomarker of metabolic disease onset.

**Table 1:** **16S rRNA relative abundance comparison between CCHC and HMP subject stool samples**

	**CCHC**	**HMP**	***P*** **value**	***q*** **value**
Phylum				
*Firmicutes*	56.7%	38.1%	<0.001	<0.02
*Bacteroides*	37.2%	57.0%	<0.001	<0.02
*Actinobacteria*	1.28%	0.28%	<0.001	<0.02
Family				
*Lachnospiraceae*	26.8%	17.6%	<0.001	<0.01
*Veillonellaceae*	3.10%	1.46%	<0.001	<0.01
*Coriobacteriaceae*	1.22%	0.23%	<0.001	<0.01
*Ruminococcaceae*	20.6%	14.3%	<0.01	<0.01
*Prevotellaceae*	12.1%	4.4%	<0.01	<0.02
*Bacteroideaceae*	17.8%	40.2%	<0.001	<0.01
*Rikenellaceae*	1.64%	5.35%	<0.001	<0.01
Genus				
*Prevotella*	11.9%	4.10%	<0.01	<0.05
*Collinsella*	1.09%	0.16%	<0.001	<0.02
*Roseburia*	1.37%	0.95%	<0.001	<0.02
*Streptococcus*	0.68%	0.05%	<0.001	<0.02
*Dialister*	0.55%	0.13%	<0.01	<0.05
*Bacteroides*	17.7%	39.8%	<0.001	<0.02
*Alistipes*	1.51%	5.10%	<0.001	<0.02
*Parabacteroides*	1.92%	4.58%	<0.001	<0.02

*P* and *q* values were calculated via metastats (http://metastats.cbcb.umd.edu/detection.html).

**Figure 5:**
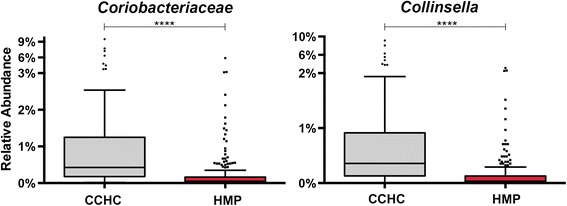
***Coriobacteriaceae*** **and** ***Collinsella*** **16S rRNA relative abundance differences between CCHC and HMP stool samples.** Tukey style box plots of the differences in 16S rRNA relative abundance of the family *Coriobacteriaceae* and the genus *Collinsella* between CCHC and HMP subject stool samples. Statistical significance was evaluated by Mann-Whitney *U* test where **** = *P* < 0.0001; HMP *n* = 213, CCHC *n* = 63. CCHC, Cameron County Hispanic Cohort; HMP, Human Microbiome Project.

In this subset of CCHC members compared to the HMP, we find significantly increased *Coriobacteriaceae*, and specifically the genus *Collinsella* (Figure 5). These shifts are strongly correlated with high low-density lipoprotein levels and high total cholesterol in both human and animal studies [43,44]. For example, the observation was made decades ago that germ-free animals have higher serum cholesterol [45]. Individuals with borderline-high total cholesterol (≥200 mg/dL) and high total cholesterol (≥240 mg/dL) are more prevalent in these CCHC study subjects compared to Mexican Americans nationwide (CCHC 50% and 22.2% versus all Mexican Americans 46.4% and 14.3%, respectively). Additionally, a higher proportion of CCHC subjects in this study have elevated LDL cholesterol (≥130 mg/dL) versus Mexican Americans nationally (CCHC 30.2% versus all Mexican Americans 27.7%). Members of the *Coriobacteriaceae* family respond to dietary interventions involving grain sorghum lipids and dietary whole grains that decrease cholesterol absorption by the host [21,46]. *Coriobacteriaceae* levels were negatively associated with improved metabolic and immunological markers after dietary intervention [21]. Similarly, studies also noted significantly higher *Collinsella* in omnivores compared to vegetarians [47] and significant reductions of *Collinsella* on a low-carb weight loss diet [48]. Together these observations show that *Collinsella* and perhaps other members of *Coriobacteriaceae* are often positively correlated with disease, particularly elevated cholesterol, and may be a worthwhile target for behavioral, probiotic, and/or prebiotic manipulation or as a diagnostic biomarker.

**Figure 6:**
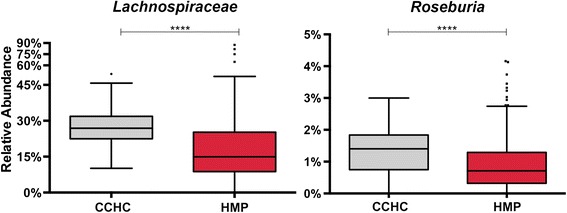
***Lachnospiraceae*** **and** ***Roseburia*** **16S rRNA relative abundance differences between CCHC and HMP stool samples.** Tukey style box plots of the differences in 16S rRNA relative abundance of the family *Lachnospiraceae* and the genus *Roseburia* between CCHC and HMP subject stool samples. Statistical significance was evaluated by Mann-Whitney *U* test where **** = *P* < 0.0001; HMP *n* = 213, CCHC *n* = 63. CCHC, Cameron County Hispanic Cohort; HMP, Human Microbiome Project.

In addition, among our study subjects, we observe a significantly higher *Firmicutes*:*Bacteroidetes* ratio compared to the HMP (Figure 4). Higher fecal concentrations of short-chain fatty acids (SCFA) in obese compared with lean individuals have been attributed to a higher *Firmicutes*:*Bacteroidetes* ratio [49]. In this study, significantly more reads were identified as *Lachnospiraceae* and *Roseburia* (*Firmicutes*) in the CCHC subjects compared to the HMP (Figure 6). Increased *Lachnospiraceae* in particular has been associated with obesity, non-alcoholic fatty liver disease (NAFLD), and protection from colorectal cancer [50,51]. The protection from colorectal cancer is attributed to higher butyrate production, an ability harbored by many species in the *Lachnospiraceae* family including species of the genus *Roseburia*. Interestingly, these observations correlate with the observation that Hispanics living along the Texas-Mexico border have significantly lower rates of colorectal cancer than those living in non-border counties [52]. In the CCHC, colorectal cancer ranks seventh amongst women and sixth amongst men in incidence, whereas colorectal cancer ranks second nationally for Hispanics of both sexes [53]. None of the subjects included in this study had colorectal cancer. Additionally, NAFLD is found at much higher rates amongst the CCHC members (46% of study subjects had elevated alanine aminotransferase), and Hispanic individuals in general [54]. These observations demonstrate the importance of understanding host/microbial relationships in order to prevent unforeseen effects of targeted gut microbial community manipulation, because gut microbial composition may be concurrently protective of and predisposing to certain diseases.

**Figure 7:**
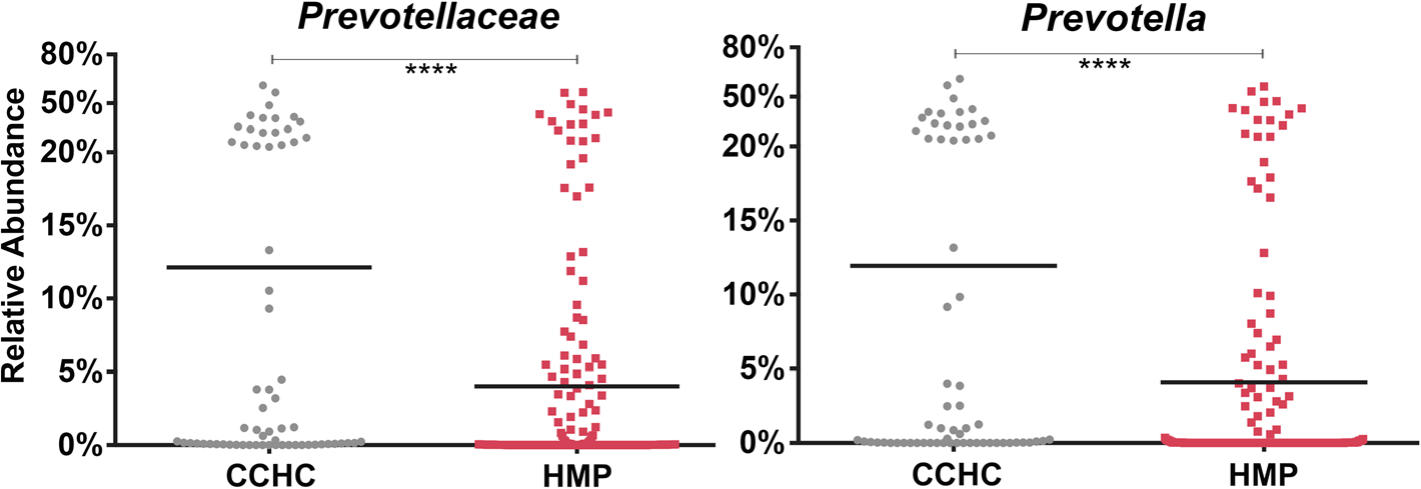
***Prevotellaceae*** **and** ***Prevotella*** **16S rRNA relative abundance differences between CCHC and HMP stool samples.** Scatter plots representing 16S rRNA relative abundance of the family *Prevotellaceae* and the genus *Prevotella*. Black bars represent mean relative abundance. All *P* values were calculated by Mann-Whitney *U* test where **** = *P* < 0.0001; HMP *n* = 213, CCHC *n* = 63. CCHC, Cameron County Hispanic Cohort; HMP, Human Microbiome Project.

The *Prevotellaceae* family has exhibited strong positive associations with obesity [55] and impaired glucose tolerance [56], while having a negative association with type 1 diabetes [57,58]. In the CCHC, subjects clustered distinctly by their relative abundance of *Prevotella* into two groups (Figure 7). Of note, Bergstrom *et al*. [19] found a bimodal distribution of *Prevotella* apparent by the third year of life, and Roager *et al*. [18] showed that, in adults, this high/low *Prevotella* grouping remained stable during a 6-month dietary intervention, even after courses of antibiotics. Numerous recent studies have linked high prevalence of this family to a predominately plant-based diet [47,59-61]. However, it seems unlikely that differences in diet are the sole explanation for the bimodal distribution of *Prevotella* within the CCHC. All of the CCHC subjects are from the same general neighborhood, are of the same ethnicity, share similar socioeconomic status, and in some cases are family members. Interestingly, family members were as often discordant for high/low *Prevotella* grouping as concordant. This suggests factors additional to diet may determine the prevalence of *Prevotella* in the gut microbial community. In the CCHC, the bimodal distribution was not explained by age, sex, BMI, waist-to-hip ratio, income quartile, birth country, total cholesterol, triglycerides, high blood pressure, diabetes status, fasting glucose, or weight change over 5 years (as percentage of body weight). This recurring observation of sample division by high/low *Prevotella* abundance remains intriguing; however, none of the metadata we examined suggests a reason for the distinct grouping.

### OTU network analysis

The gut microbial environment is influenced by host genetics, host diet, and other factors and may influence the way the gut microbiota interact with each other and the host [20,62]. We sought to determine whether OTUs exhibited correlative associations across individuals in the CCHC, possibly revealing biologically relevant relationships between taxa not discernable from other types of analysis. Among other applications, this information can prove useful for *in vitro* manipulation of organisms currently recalcitrant to culture [63,64] and will likely be indispensible as probiotics and personalized medical treatments are developed.

**Figure 8:**
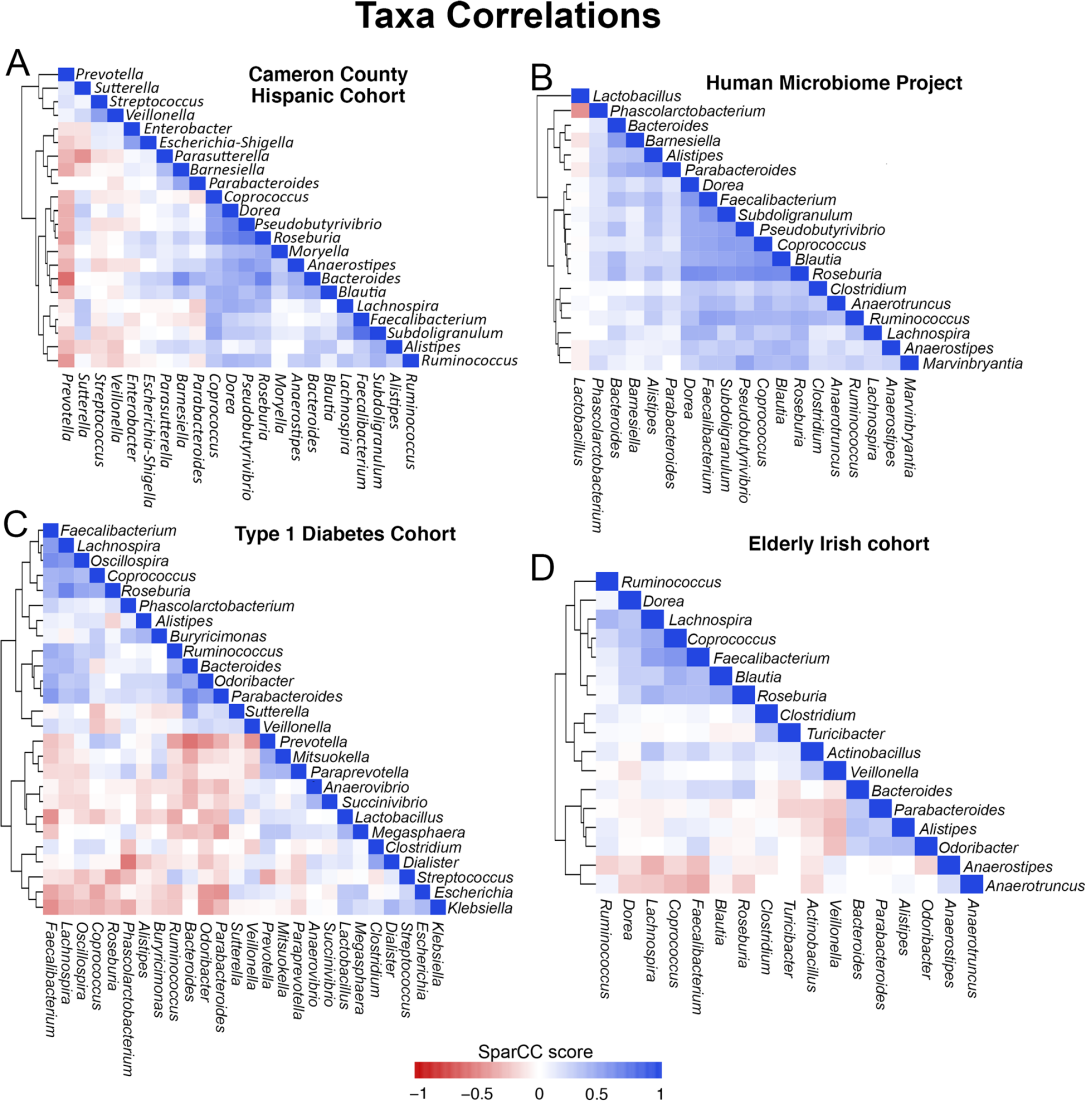
**Heat maps showing the strength of OTU correlations calculated by the SparCC algorithm.** These heat maps represent the strength of pairwise OTU-OTU co-occurrence/co-exclusion calculations made by the SparCC algorithm. Scores range from −1, a perfect negative correlation, to 1, a perfect positive correlation. **(A)** Cameron County Hispanic Cohort subjects. **(B)** Human Microbiome Project subjects. **(C)** Type 1 diabetes cohort subjects. **(D)** Elderly Irish cohort subjects. For **(A)**, **(B)**, and **(C)** only OTUs with a correlation above 0.4 were plotted for clarity. For **(D)**, OTUs with a correlation above 0.3 were plotted.

**Figure 9:**
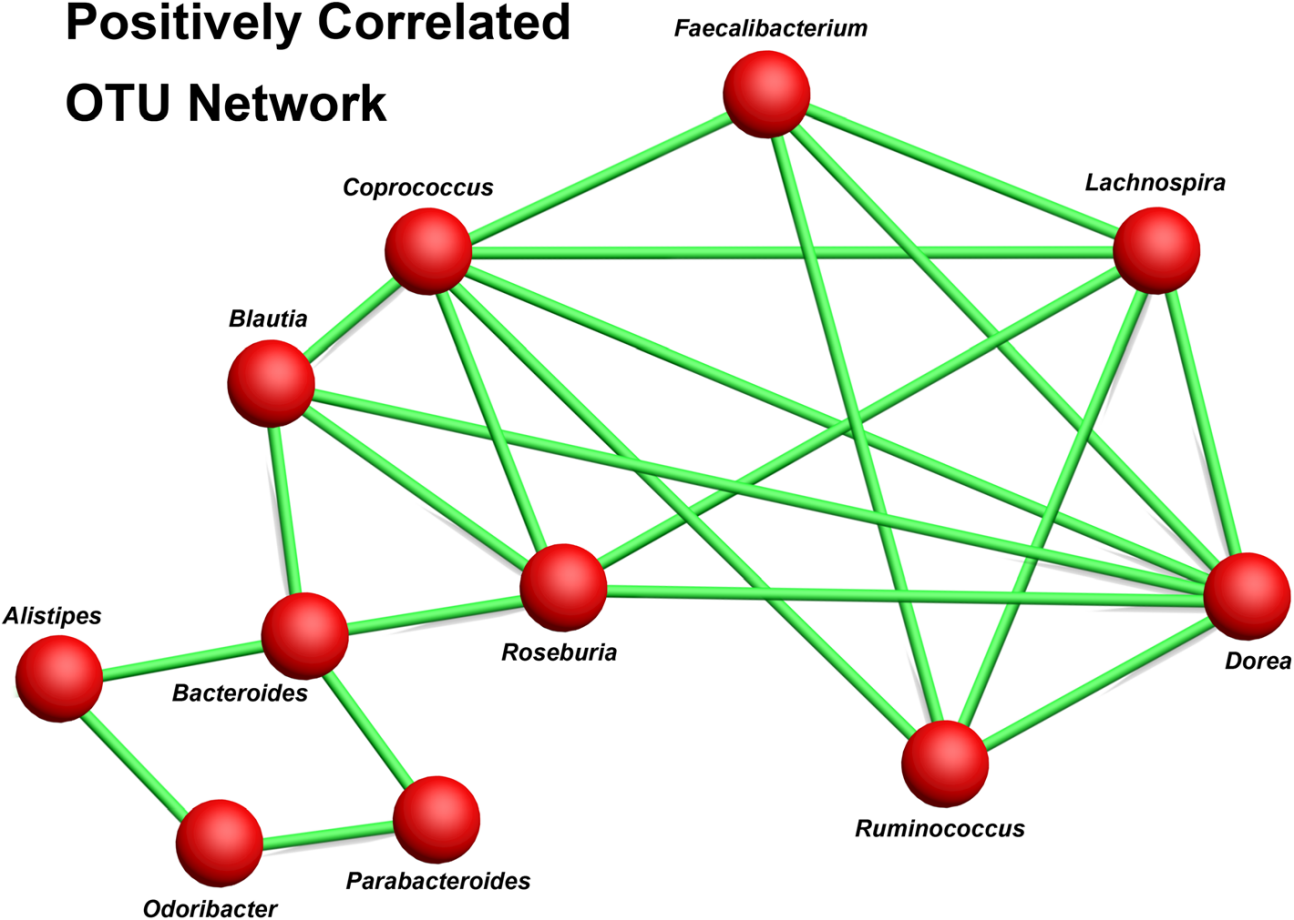
**Network plot of commonly positively correlated taxa.** This edge/node style plot shows OTU-OTU co-occurrence correlations that appeared in at least three of the four datasets tested. Nodes (red spheres) represent genera and edges (green lines) represent a correlation across multiple datasets. OTU, operational taxonomic unit.

We incorporated two additional, unrelated datasets to diversify the population for the OTU correlation analysis to determine whether correlated OTUs could either remain so across diverse populations, age groups, and socioeconomic backgrounds or whether these correlations may only hold true for certain population strata. These additional datasets include a type 1 diabetes cohort from Mexico [57] and an elderly cohort from Ireland [65] (Figure 8). We identified 22 OTU pairs that were positively correlated in at least three of the four datasets, suggesting a mutualistic relationship or possibly codependency (Figure 9). Additionally, these 22 OTU pairs only comprise 11 distinct genera, with only 7 genera contained in 18 of the pairings. This highlights a small network of highly correlated OTUs present in the human gut. Of these seven highly correlated OTUs, five belong to the butyrate-producing family *Lachnospiraceae*, while the remaining two belong to *Ruminococcaceae.* Of these seven genera, all but one, *Lachnospira*, were significantly increased in the CCHC compared to the HMP. Many of these genera are noted for containing species that produce butyrate through fermentation of hydrolyzed polysaccharides and may explain the low rates of colon cancer seen in this population. Additional studies have observed depletion of these genera in T2D, suggesting that diminished butyrate production in the gut may play a role in the pathogenesis of T2D [66].

Negative correlations were less abundant and no OTU pairs were negatively correlated across all four datasets; however, there were nine pairs that were negatively correlated in two or three of the datasets. Many studies have noted a strong co-exclusive relationship between *Prevotella* and *Bacteroides*; however, these were only strongly negatively correlated in two of the four datasets (CCHC and T1D). In the two datasets in which there was little correlation between *Prevotella* and *Bacteroides* (HMP and elderly), *Prevotella* was present at very low abundance across subjects. This suggests that the host environment, such as differences imparted by genetic factors or by diet, may tip the balance in a competition between species as has been suggested for *Prevotella*. In both the T1D (children) and elderly cohorts, we found that strong OTU correlations, both positive and negative, were much less abundant. This may be explained by the increased flux in the microbiota in the very young and the very old as well as extreme interpersonal variability in these age groups [65,67].

## Conclusions

Within the CCHC, there are no significant gut microbiome shifts associated with age, sex, disease status, or any other available measure. This suggests that the often-reported ‘disease’ associated gut community shifts we found in the CCHC manifest early and likely persist for life. The youngest CCHC member of this analysis set is 28 years old, thus sampling this population at earlier time points would provide insight into the age at which these observed signatures become apparent. Whether causal or an effect of outside influences, this persistent community structure might compound other predisposing genetic or lifestyle factors, leading to the higher rates of obesity, T2D, NAFLD, and other diseases observed in the CCHC. However, this gut community structure may contribute to the low rates of colorectal cancer observed in the CCHC through increased production of butyrate, suggesting that a particular gut microbiome composition can be both predisposing and protective of different diseases simultaneously. This highlights the need for a more complete understanding of host/microbe relationships when implementing targeted manipulation of the human gut community.

Among other observations, we identified a core group of taxa that appear to be tightly correlated across many populations. A large portion of these taxa belong to the butyrate-producing family *Lachnospiraceae* and were found to be increased in relative abundance in the CCHC compared to the HMP. This correlated group of taxa is also often depleted in T2D patients, suggesting a possible link between the metabolic functions of these taxa and progression of T2D. Information about microbial codependence will be indispensable in the creation of synthetic communities for probiotics or other purposes.

Manipulation of the intestinal microbiome shows promise as a therapy for many diseases; nonetheless, a comprehensive understanding of the mechanisms of action remains to be deciphered. Members of the CCHC exhibit increased prevalence of obesity and T2D, which make them an interesting population to utilize for unraveling related host/microbiome relationships. However, this was a cross-sectional study and thus lacks the resolution necessary to elucidate how the microbiome signatures in the CCHC specifically impact these diseases.

Additionally, we found a higher level of gut microbiome homogeneity within the CCHC compared with other sampled populations such as the HMP (average variance between CCHC subjects across phyla was more than threefold lower than HMP subjects), perhaps making the CCHC an ideal community to test prebiotic or probiotic interventions. Future directions will include longitudinal sampling of CCHC members so that we may assess the low variability and overall stability of the gut microbiome signatures observed in this cohort and its involvement in disease states.

## Methods

A subset of participants from the original CCHC study [35] were re-solicited for a follow-up visit and asked to provide a stool sample. This follow-up visit occurred approximately 5 years after the original study visit. Stools were self-collected at home, delivered to the clinic, aliquoted into 50 mL tubes, mixed to a 50% suspension in RNAlater (Invitrogen, Carlsbad, CA, USA) and stored at −20°C within 24 h of collection. A total of 69 stool samples were delivered to Baylor College of Medicine for extraction and sequencing, and sequence data was generated for 63 of them. Samples for both the HMP and CCHC studies were processed via the same protocols [39] and, in most cases, by the same personnel. The samples were thawed, vortexed, and a wide-bore tip was used to transfer approximately 750 μL of the slurry to a MoBio PowerSoil garnet bead tube (Mo Bio Laboratories, Carlsbad, CA, USA). The stool was processed according to the kit instructions and eluted into 50 μL. 16S rRNA sequencing was performed on the V1 to V3 region (primers 27f and 534r) and sequenced via Roche 454 pyrosequencing (454 Life Sciences, Branford, CT, USA) (average 12,900 reads/sample). Raw fastq files have been deposited into SRA (project accession SRP053023). Sequences were processed using QIIME software [68]. Reads were quality trimmed using default settings and normalized to 3,100 reads/sample. All CCHC clinical metadata are included in Additional file 1. OTUs were picked closed-reference using uclust-ref against the Silva database release 111 at 97%. OTU network correlation analysis was performed using 16S rRNA relative abundance data to identify clusters of highly correlated OTUs. Iterations of the sparse correlations for compositional data (SparCC) algorithm [69] were used to generate correlation matrices in this analysis. This algorithm is specifically designed to account for compositional effects often present in genomic survey data, such as 16S rRNA sequence data. We chose the HMP dataset to compare against, as this represents a standard gut microbiome of a healthy population [39-41]. For our analysis, we used all HMP stool samples that had 16S rRNA V1 to V3 region data generated and also contained at least 3,100 reads, which amounted to 213 samples.

## Additional file

Additional file 1:
**CCHC clinical metadata.** This file contains relevant clinical metadata on the subjects included in this study.
